# Proteomic Insights into the Retinal Response to PRGF in a Mouse Model of Age-Related Macular Degeneration

**DOI:** 10.3390/medicina61122235

**Published:** 2025-12-18

**Authors:** Eduardo Anitua, Francisco Muruzabal, Sergio Recalde, María de la Fuente, Iraia Reparaz, Mikel Azkargorta, Félix Elortza, Mohammad Hamdan Alkhraisat

**Affiliations:** 1 Regenerative Medicine Laboratory, BTI—Biotechnology Institute, 01007 Vitoria, Spain; francisco.muruzabal@bti-implant.es (F.M.); maria.delafuente@bti-implant.es (M.d.l.F.); iraia.reparaz@bti-implant.es (I.R.); mohammad.hamdan@bti-implant.es (M.H.A.); 2 University Institute for Regenerative Medicine and Oral Implantology—UIRMI (UPV/EHU-Fundación Eduardo Anitua), 01007 Vitoria, Spain; 3 Retinal Pathologies and New Therapies Group, Experimental Ophthalmology Laboratory, Department of Ophthalmology, Clínica Universitaria de Navarra, 31008 Pamplona, Spain; srecalde@unav.es; 4 Navarra Institute for Health Research, IdiSNA, 31008 Pamplona, Spain; 5 Proteomics Platform, Center for Cooperative Research in Biosciences (CIC bioGUNE), Basque Research and Technology Alliance (BRTA), 48160 Derio, Spain; mazkargorta@cicbiogune.es (M.A.); felortza@cicbiogune.es (F.E.); 6 Department of Oral and Maxillofacial Surgery, Oral Medicine and Periodontology Faculty of Dentistry, University of Jordan, Amman 11942, Jordan

**Keywords:** age-related macular degeneration (AMD), geographic atrophy (GA), platelet rich plasma (PRP), plasma rich in growth factors (PRGF), retina, retinal pigment epithelial cell, animal model

## Abstract

*Background and Objectives*: The aim of this study is to employ quantitative proteomics to elucidate the molecular mechanism and signaling pathways modulated by plasma rich in growth factors (PRGF) in a murine model of geographic atrophy (GA)-like retinal degeneration. *Materials and Methods*: C57BL/6J mice were used as a model GA-like retinal degeneration by a single systemic NaIO_3_ administration. Animals were divided into three groups: Control (PBS), Disease (NaIO_3_ + PBS), and PRGF-treated (NaIO_3_ + PRGF). After 7 days, retinas and retinal pigment epithelium were collected for proteomic analysis. Proteins were extracted, digested using the FASP method, and analyzed by Data-Independent Acquisition (DIA-PASEF) mass spectrometry; data were processed with DIA-NN and statistically analyzed with Perseus. Functional pathway analysis was performed using Ingenuity Pathway Analysis. *Results*: A total of 6511 proteins were identified. The Disease model showed the expected deregulation of pathways related to oxidative stress, inflammation, and fibrosis. Comparison between the PRGF and Control groups showed that PRGF significantly reduced oxidative and cellular stress proteins/pathways. In the same way, when PRGF and Disease groups were compared, PRGF treatment showed a significant reduction in pathways associated with inflammation, oxidative stress, and cellular stress. PRGF also activated several homeostatic pathways not only related to neuroprotective pathways but also with the lipid deposition (drusen) reduction. All these results suggest that PRGF treatment exerts a protective effect against NaIO_3_-induced retinal damage. *Conclusions*: These findings suggest that PRGF effectively mitigates the degenerative effects of NaIO_3_ by activating specific protective and compensatory signaling pathways in the retina. PRGF is indicated as a promising new therapeutic option for ameliorating age-related macular degeneration progression.

## 1. Introduction

Age-related macular degeneration (AMD) is a chronic, multifactorial retinal disease and the leading cause of irreversible vision loss in adults worldwide. In 2020, AMD was responsible for blindness in approximately 1.85 million individuals and moderate-to-severe visual impairment in an additional 6.23 million [1]. By 2040, the global prevalence is projected to reach 288 million cases [2]. Its incidence increases markedly with age, being influenced by both genetic susceptibility [3,4] and environmental exposures [5]. AMD initially compromises the central area of the retina (macula), leading to the deterioration of the central vision and impairing fine detail perception. AMD is classified into three stages: early, intermediate, and late stage (advanced AMD). Late AMD is generally classified into two groups, dry AMD or geographic atrophy (GA) and wet or neovascular AMD (nAMD), which may coexist in the same patient or eye [6]. The dry form accounts for approximately 90% of cases and includes a spectrum ranging from early drusen accumulation and RPE irregularities to geographic atrophy. GA is defined by well-demarcated areas of progressive degeneration of the RPE, photoreceptors, Bruch’s membrane, and underlying choriocapillaris, ultimately resulting in irreversible scotomas that enlarge over time [7].

The pathogenesis of AMD involves interconnected mechanisms, including oxidative stress, chronic inflammation, complement system dysregulation, extracellular matrix remodeling, lipid accumulation, and, in advanced stages, leads to irreversible atrophy of the retinal pigment epithelium (RPE), choriocapillaris, and photoreceptor cells in the macular region [8,9,10,11].

Despite extensive research efforts, there is currently no approved therapy that slows the progression of early or intermediate-to-late AMD nor is there a therapy that halts or reverses geographic atrophy (GA) development. This crucial unmet need has led to a focus on regenerative and neuroprotective strategies to preserve the integrity and function of the outer retina. Current investigational approaches include complement inhibitors, anti-inflammatory agents, gene therapies, and stem cell-based interventions targeting RPE preservation or replacement [12,13,14].

Among emerging strategies, platelet-rich plasma (PRP) and other platelet-derived formulations have garnered attention due to their high concentration of growth factors, cytokines, and antioxidants, which possess the potential to provide neuroprotective benefits and promote tissue homeostasis [15,16]. Cord blood-derived PRP (CB-PRP) has been explored through subretinal [17] and intravitreal [18,19] administration. Early-phase clinical studies have demonstrated a favorable safety profile, with no serious ocular or systemic adverse events [17,18,19]. In the subretinal trial, structural benefits included reduced autofluorescence progression and qualitative preservation of the outer retinal layers [17]. Intravitreal studies reported suggestive trends toward stabilization of the GA area and outer retinal thickness, although without statistically significant improvement in visual acuity [18,19]. These findings highlight the potential of CB-PRP as a safe, biologically active adjunctive therapy for atrophic AMD, warranting further controlled trials to confirm efficacy.

Plasma rich in growth factors (PRGF) is defined as a leukocyte-free PRP formulation obtained via standardized centrifugation and calcium-induced activation [20]. In contrast to conventional PRP, PRGF minimizes inflammatory risk by excluding proinflammatory leukocytes while also providing controlled growth factor release [21]. In a recent study, the administration of a single intravitreal injection of PRGF in a sodium iodate-induced murine model of retinal degeneration, which mimics key features of GA, induced a significant retinal neuroprotection, inducing a reduction in retinal thinning and outer nuclear layer disruption, while attenuating microglial activation and inflammatory marker expression [22]. However, the molecular mechanisms underlying these protective effects remain poorly defined.

Building on these findings, the present study applies a proteomic approach to elucidate the molecular mechanisms by which PRGF modulates retinal degeneration. Here, we used the systemic administration of sodium iodate (NaIO_3_) as an AMD murine model, inducing a focal atrophic area that simulated human geographic atrophy. The oxidizing chemical sodium iodate (NaIO_3_) causes necroptosis of RPE cells and damage to adjacent cell layers through oxidative stress-related processes [23,24]. After inducing damage, an intraocular injection of PRGF was performed in the AMD mouse model. The response was then analyzed at the protein level by obtaining the retinas and RPE. The main objective of the present study was to identify the key signaling pathways and cellular processes regulated by PRGF by comparing the proteomic profiles of healthy, GA-like, and PRGF-treated murine retinas and RPE. This analysis may provide critical mechanistic insights into the regenerative and neuroprotective actions of PRGF, potentially guiding the development of novel therapeutic strategies for geographic atrophy.

## 2. Materials and Methods

The study was conducted in accordance with the European Community guidelines for the ethical animal care and use of laboratory animals (Directive 2010/63/UE) and received approval from the University of Navarra Animal Research Review Committee (47E2022). Male and female C57BL/6J mice (Charles River, Wilmington, MA, USA) aged 8 to 12 weeks were used for this study. The mice were raised under controlled lighting conditions (12:12 light–dark cycle) with ad libitum access to standard food and water.

### 2.1. Experimental AMD Animal Model and Study Treatments

The animal groups, experimental procedures, and PRGF treatment used in this study were identical to those previously described in our earliest publication focused on the structural and immunological retinal effects of PRGF in a murine model of AMD [22].

Briefly, mice were first anesthetized by an intramuscular injection of anesthetic mixture composed of 80 mg/kg of ketamine (Daiich-Sankyo, Tokyo, Japan) and 6 mg/kg of xylazine (Bayer, Health Care, Osaka, Japan); then, they received intraperitoneal injections of 40 mg/kg of sodium iodate (NaIO_3_) to induce the retinal pathology. On the other hand, control animals were injected with an equivalent volume of phosphate-buffered saline (PBS). Immediately after systemic administration, a single intravitreal injection of PRGF supernatant (treatment group) or PBS (Control group) was carried out. Then, the study was conducted using 3 experimental groups of mice: (i) Control group, receiving PBS injection both intramuscularly and intraocularly; (ii) Disease model group, induced by NaIO_3_ intramuscular injection and a PBS intraocular injection; and (iii) PRGF group, which received NaIO_3_ intramuscular injection and was subsequently treated intraocularly with a PRGF injection.

### 2.2. PRGF Preparation

PRGF was obtained from pooled whole blood collected from six C57BL/6 donor mice (three males and three females), anesthetized and euthanized by CO_2_ inhalation. Blood was drawn via intracardiac puncture into 2.7 mL tubes containing 3.2% sodium citrate (*w*/*v*) as an anticoagulant. The samples were then centrifuged at 400× *g* for 8 min, and the plasma fraction above the buffy coat was collected. This plasma was activated by adding calcium chloride and incubated at 37 °C for 1 h to promote platelet degranulation and thus the release of growth factors. The resulting supernatant was filtered through a 0.22 μm PES membrane to obtain the PRGF formulation used in the study.

### 2.3. Retinal Tissue Collection

After 7 days of treatment, mice were anesthetized prior to the euthanasia process using a CO_2_ gradient. Eyes were enucleated using specialized surgical instruments. The retina and RPE were carefully dissected under a stereomicroscope to ensure anatomical integrity. Immediately after dissection, tissues were frozen and stored at −20 °C until further processing for proteomic analysis.

### 2.4. Protein Extraction

For protein extraction, each sample was incubated with 200 μL of RIPA buffer (RIPA Lysis Buffer, Sigma-Aldrich Corp., St. Louis, MO, USA) supplemented with protease inhibitors (Complete Mini EDTA-free, Roche, Basilea, Switzerland) and phosphatase inhibitors (PhosSTOP EASYpack, Roche, Basilea, Switzerland). Tissue homogenization was performed using the Bullet Blender Tissue Homogenizer Storm 24 (Next Advance, Troy, NY, USA) with RNase-free zirconium oxide beads (0.5 mm diameter, Next Advance, Troy, NY, USA), ensuring efficient mechanical disruption under cold conditions. Homogenates were centrifuged at 13,000 rpm for 30 min at 4 °C. The resulting supernatants were collected and stored at −20 °C until further analysis.

### 2.5. Sample Preparation

Samples were digested following the FASP protocol described by Wisniewski et al. [25] with minor modifications. Trypsin was added in 50 mM ammonium bicarbonate to a trypsin–protein ratio of 1:10, and the mixture was incubated overnight at 37 °C. Peptides were dried out in an RVC2 25 SpeedVac concentrator (Christ, Osterode am Harz, Germany) and resuspended in 0.1% formic acid (FA). Peptides were desalted and resuspended in 0.1% FA using C18 stage tips (Millipore, Burlington, MA, USA) prior to acquisition.

### 2.6. Mass Spectrometry Analysis

The resulting peptides were loaded onto an EvoSep One (EvoSep, Odense M, Denmark) chromatograph coupled on-line to a TIMS ToF Pro mass spectrometer (Bruker, Karlsruhe, Germany) that uses Parallel Accumulation Serial Fragmentation (PASEF) acquisition to provide extremely high speed and sensitivity. The 30 SPD protocol (approx. 44 min runs) was used under default Evosep settings. Data-Independent Acquisition (DIA) was used for the acquisition of data.

### 2.7. Protein Identification and Quantification

DIA data was processed with DIA-NN [26] software (version 1.8.1) for protein identification and quantification using default parameters. Searches were carried out against a database consisting of Mus musculus protein entries from UniProt in library-free mode. Carbamidomethylation of cysteines was considered as fixed modification and oxidation of methionines as variable modification. Data was loaded onto Perseus platform [27] for data processing (log2 transformation, selection of proteins detected in at least the 70% of the samples in at least one of the groups, imputation) and statistical analysis (Student’s *t*-test). Proteins with a *p* < 0.05 were considered for further analyses and discussions.

### 2.8. Functional Analysis

Ingenuity Pathway Analysis (IPA, QIAGEN Redwood City, www.qiagen.com/ingenuity (accessed in October 2025)) was used for a characterization of the molecular events lying behind the differential protein patterns under analysis. In this software, the calculated *p*-values determine the probability that the association between proteins in the dataset and a given process, pathway, or upstream regulator is explained by chance alone, based on Fisher’s exact test (*p*-value < 0.05 being considered significant). The activation z-score represents the bias in gene regulation that predicts whether the upstream regulator exists in an activated (positive values) or inactivated (negative values) state, based on the knowledge of the relation between the effectors and their target molecules. Only significantly enriched (*p* < 0.05) and theoretically modulated (Z score > 2 or <−2) pathways or regulators were considered for the discussion

## 3. Results

### 3.1. Protein Profile Evaluation

**Figure 1:**
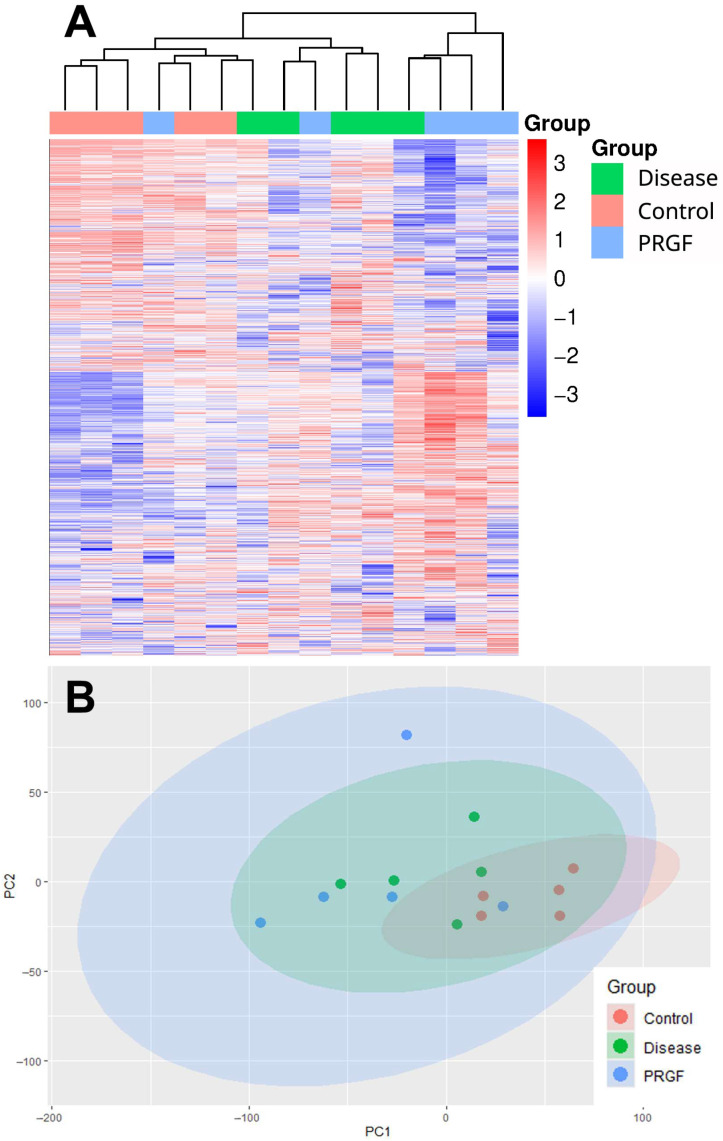
Differential protein expression of the different mice included in different groups (Control, Disease, and PRGF; *n* = 5 for each group). (**A**) Heatmap of the analysis of differentially expressed proteins in the retina of each mouse. The red color represents higher z-scores and, therefore, higher relative abundances for each protein. The blue color represents proteins with lower relative abundances and lower z-scores. (**B**) Principal component analysis plot showing the clustering of the protein profiles from the different mice belonging to the different groups. The protein profiles of the samples can be clustered into two main groups: one containing mainly mice from the Control group and another containing mice treated with NaI03 (Disease and PRGF groups).

Protein profiles from the three conditions (Control, Disease, and PRGF) were obtained (Supplementary File S1). A total of 6511 proteins were detected in the different samples. A heatmap for all proteins was drawn to analyze the clustering of the protein profile obtained from each mouse belonging to each treatment group (Figure 1A). Although the protein profile of some individuals in the PRGF group is similar to that of other groups, the heatmap representation showed that the protein profile provided by each mouse may mainly be grouped into the three treatment groups (Control, Disease, and PRGF). However, the principal component analysis (PCA) scatter plot (Figure 1B) shows that the protein profiles of the different mice could be grouped into two main clusters, one mainly containing mice from the Control group and the other containing mice treated with NaI0_3_ (Disease and PRGF groups).

### 3.2. Differential Protein Analysis

**Figure 2:**
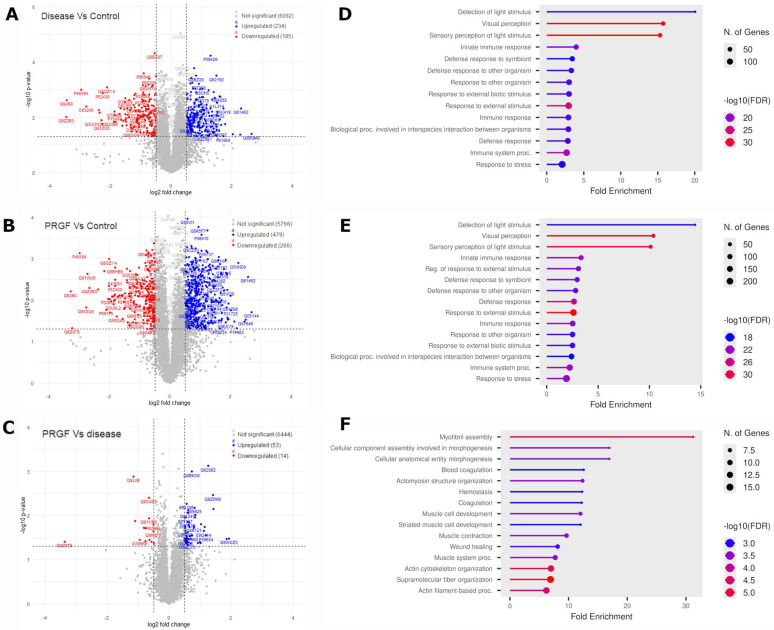
Analysis of the deregulated proteins obtained after comparing the different groups. (**A**–**C**) Volcano plot illustrating differentially regulated proteins in (**A**) Disease vs. Control, (**B**) PRGF vs. Control, and (**C**) PRGF vs. Disease. Upregulated proteins (log2 Fold Change > 0.5 and *p* < 0.05) are represented as red dots, while downregulated proteins (log2 Fold Change < −0.5 and *p* < 0.05) are represented as blue dots. Spots located further from the center indicate that the proteins exhibit the greatest change in expression between the groups. (**D**–**E**) results of the GO enrichment analysis performed using the ShinyGO application (version 0.85.1). Each plot represents the deregulated biological processes identified in each comparison: (**D**) Disease vs. Control, (**E**) PRGF vs. Control, and (**F**) PRGF vs. Disease. Each plot shows the top hallmark pathways, where spot size represents the number of differentially expressed proteins belonging to the pathway, and spot color indicates the confidence enrichment (FDR). X-axis denotes the enrichment factor of differentially expressed proteins.

Differential protein expression was analyzed across the three conditions: Control, Disease, and PRGF (Figure 2). The analysis was carried out between the different conditions: Disease vs. Control, PRGF vs. Control, and PRGF vs. Disease (Supplementary File S2). Only proteins with at least two non-conflicting peptides that achieved statistical significance (*p* < 0.05) and displayed a Fold Change (ratio) higher than 1.5 in any comparison were chosen for downstream analysis. The differential protein analysis revealed different distributions of up- and downregulated proteins across the three comparison groups (Figure 2A–C). In the Disease vs. Control group, 234 upregulated and 185 downregulated proteins were detected, totaling 419 proteins (Figure 2A). Conversely, the PRGF vs. Control comparison yielded the highest overall deregulation, identifying 479 upregulated and 266 downregulated proteins for a total of 745 proteins (Figure 2B). Finally, the final comparison of PRGF vs. Disease showed the lowest level of alteration, revealing only 53 upregulated and 14 downregulated proteins (67 total) (Figure 2C).

Gene Ontology (GO) analysis was carried out on the deregulated proteins observed in each comparison (Disease vs. Control, PRGF vs. Control, and PRGF vs. Disease) to broadly characterize the functional processes these proteins are involved in (Figure 2D–F). Enrichment was determined using Fisher’s exact test. Values of *p* < 0.05 were selected for each of the protein lists and subsequently compared (Supplementary File S2). When the 20 most representative biological processes of the deregulated proteins in the Disease and PRGF groups were evaluated in comparison with the Control group, it was observed that they showed a similar profile but exhibited statistically significant differences in their values. (Figure 2D,E). The deregulated proteins related to these biological processes can be grouped into three main biological functions: light detection, immune response, and cellular stress. This may indicate a severe failure in visual function (damaged photoreceptors) combined with active neuroinflammation and an intense stress response. These findings may be related to the toxic effect on the retina induced by the NaIO_3_ in both Disease and PRGF groups in comparison with the Control group. However, the GO terms obtained after comparing PRGF with Disease could be grouped into five key functions: muscle development, cytoskeletal organization, blood coagulation, inflammatory response, and cell morphogenesis (Figure 2F).

However, the deregulated proteins observed in the comparison between the PRGF and Disease groups revealed GO terms related to processes essential for visual function and responses to tissue damage. These GO terms may be grouped into three main processes: (i) muscle development and cytoskeletal organization, (ii) coagulation and inflammation, and (iii) cell morphogenesis and structural assembly. Muscle development and cytoskeletal organization are related to the presence of cells with a contractile ability, like myofibroblast, which may be present in the choriocapillaris tissue after retinal damage or may form part of the epiretinal membrane. On the other hand, coagulation and inflammation suggest protective and reparative mechanisms that are activated in response to an injury or oxidative stress after retinal tissue damage. Finally, cell morphogenesis and structural assembly are fundamental to the development and maintenance of the retinal structure, including photoreceptors and supporting cells.

### 3.3. Ingenuity Pathway Analysis

Ingenuity Pathway Analysis (IPA) was carried out for further characterization of the functional processes in which the proteins with significant differential expression were involved. The comparison of protein expression among the different groups (Control, Disease, and PRGF) showed that several pathways were significantly deregulated. The comparison between deregulated proteins in the PRGF and Control groups showed a total of 156 deregulated canonical pathways, consisting of 106 upregulated and 50 downregulated pathways. Finally, 52 deregulated canonical pathways (12 upregulated and 40 downregulated) were found when PRGF and Disease groups were compared (Supplementary File S3). Based on the lists, a more exhaustive analysis of the 25 most deregulated pathways observed in each comparison between the different groups was carried out (Table 1). Regarding the Disease and Control comparison, these deregulated pathways can be organized into seven main biological processes, all of which are involved in the development of degenerative retinal disease such as AMD. Five of these processes directly contribute to the pathogenesis of AMD-like oxidative stress (three pathways), cellular stress (three), angiogenesis (two), fibrosis (two), and inflammation (three). The next two are related to metabolic processes which are deregulated during AMD progression, like the increase in protein metabolism (six pathways) and the reduction or inactivation of energy metabolism (three). The results obtained in the comparison between the Disease and Control groups are consistent with the development of a mice AMD model obtained after NaIO_3_ systemic administration.

Conversely, when comparing the PRGF and Control groups, the 25 most significant canonical pathways showed that PRGF is not able to mitigate several biological processes associated with NaIO_3_-induced AMD development such as inflammation (eight activated pathways) and fibrosis (eight) (Table 2). Nevertheless, PRGF successfully reduced other pathways that are also closely related to AMD progression, such as oxidative stress (one), cellular stress (one), and energy metabolism (one). In addition, PRGF even induced the activation of four pathways related to retinal homeostasis, suggesting that PRGF treatment could reduce the degenerative effect of NaIO_3_ on retinal tissue.

When comparing the PRGF group with the Disease group, the most dysregulated biological processes were found to be upregulated in the retinas of mice in the Disease group (Table 3).

These highly upregulated pathways are mainly involved in the different functional processes mentioned as being associated with AMD progression: inflammation, oxidative stress, cellular stress, energy metabolism, and protein metabolism. The results obtained in this comparison showed that some of these functional processes related to the progression of AMD, such as inflammation, oxidative stress, and cellular stress, were completely reduced in the PRGF group compared to the Disease group. Conversely, several pathways related to angiogenesis (Semaphorin Neuronal Repulsive Signaling Pathway, Ephrin Receptor Signaling, and Regulation of Insulin-like Growth Factor (IGF) Transport and Uptake by IGFBPs) and fibrosis (including Calcium Signaling; Dilated Cardiomyopathy Signaling Pathway; Hepatic Fibrosis Signaling Pathway; Dopamine-DARPP32 Feedback in cAMP Signaling; Smooth Muscle Contraction; Nuclear Cytoskeleton Signaling Pathway; and Formation of Fibrin Clot) were found to be deregulated in both groups. These results suggest that both processes (angiogenesis and fibrosis) are in an equilibrium state in both groups, where an increase in the specific pathway could potentially shift the balance toward one side or the other of both processes. However, two pathways upregulated in the retina of the Disease groups are not only related to a fibrotic process but also to a RPE degeneration process (Dopamine-DARPP32 Feedback in cAMP Signaling) and to lipofuscin accumulation (Calcium Signaling). Both processes are strongly implicated in the development and progression of AMD. Additionally, some deregulated pathways involved in retinal homeostasis were also found in both groups: Protein Kinase A Signaling and Opioid Signaling, in the case of the Disease group, and LXR/RXR Activation and DHCR24 Signaling Pathway in the PRGF group. Overall, these findings suggest that PRGF treatment effectively reduces the degenerative effects on the retina induced by the systemic application of NaIO_3_.

## 4. Discussion

The aging population has driven the increase in age-related macular degeneration (AMD) prevalence. Advanced AMD is mainly divided into two forms: dry AMD, characterized by the retinal drusen formation and accumulation, which leads to progressive degeneration of the RPE and photoreceptors, and wet AMD, which is characterized by the abnormal proliferation of the coriocapilaris blood vessels that grow underneath the retina and macula. These vessels leak fluid or blood, causing the rapid and significant loss of central vision [28].

In contrast to neovascular AMD, which is treated by specific VEGF inhibition, dry AMD has no effective therapy, and their treatment may require a pleiotropic intervention aimed at neuroprotection and modulating RPE survival/apoptosis [29]. Research efforts are concentrating on the delivery of neurotrophic factors (e.g., GDNF, BDNF, and NGF) to promote neuronal viability and retinal synaptic plasticity [30,31]. In addition, several growth factors are involved in the response to oxidative stress and the progression of dry AMD. While some growth factors, like VEGF and TGF-β, are associated with processes such as inflammation and oxidative stress, which worsen retinal damage, others, such as PEDF, FGF, and PDGF, have a counterbalance action, contributing to cell survival and proliferation by inducing potent antioxidant and anti-inflammatory activity [32,33]. Consequently, targeting growth factors may represent a therapeutic strategy to restore retinal homeostasis, which could effectively slow AMD progression and preserve patient vision.

Plasma rich in growth factors (PRGF), a type of plasma-rich plasma, is rich in different growth factors like NGF, FGF, and PDGF, which may have effective treatment for enhancing tissue regeneration and providing neuroprotection [34,35]. Recent in vitro studies have demonstrated that PRGF exerts a significant cytoprotective effect against oxidative stress damage induced by blue light exposure [36]. The reduction in Reactive Oxygen Species (ROS) synthesis, the preservation of mitochondrial function, and restoring the PEDF/VEGF counterbalance has been proposed as the mechanism of action of the PRGF treatment [37]. In a recent study published by our group, an AMD murine model induced by systemic administration of NaIO_3_ was used to evaluate the potential of PRGF treatment to attenuate the progression of the neurodegenerative disease [22]. The results observed in that study showed that PRGF induced significant retinal neuroprotection, inducing a reduction in retinal thinning and outer nuclear layer disruption, while attenuating microglial activation and inflammatory marker expression. The present study has been carried out to identify those factors and pathways through which PRGF exerts its neuroprotective effect on retinal tissues.

As is well described in the literature, AMD pathogenesis involves several interconnected mechanisms, such as oxidative stress, chronic inflammation, complement system dysregulation, extracellular matrix remodeling, and lipid accumulation [8,9,11,38]. In addition, the oxidative stress generated in AMD induces damage in mitochondrial proteins, lipids, and DNA (mtDNA), driving mitochondrial dysfunction contributing to the increase in the oxidative stress in a self-perpetuating cycle [39]. It has also been observed that the mitochondrial damage results in the disruption and disorganization of mitochondrial cristae [40]. Furthermore, these damaged mitochondria should be degraded by a selective autophagy process denominated Mitophagy. However, this process is also dysregulated in AMD, leading to the accumulation of dysfunctional mitochondria and their components such as lipids and proteins in RPE cells [41]. Finally, AMD is also characterized by a breakdown of the intense protein metabolism (denominated Proteostasis), which is usually carried out in RPE cells. This imbalance involves both the over-accumulation of damaged and misfolded proteins and a dysregulation of key proteins involved in inflammation and the visual cycle [42].

The results observed in the present study showed that most processes involved in AMD development were dysregulated when comparing the Disease and Control groups. The most representative of these deregulated processes included oxidative stress, inflammation, different pathways related to protein metabolism deregulation, and those associated with extracellular matrix remodeling and fibrotic processes. In addition, some deregulated pathways suggest that mitochondrial dysfunction has also occurred in the retinas of diseased mice compared to those in the Control group. All these findings suggest that the mouse model obtained by the systemic administration of NaIO_3_ mimics the characteristics of AMD and is therefore suitable for evaluating the effect of PRGF treatment.

On the other hand, protein expression in the retinas of mice treated with PRGF was compared with that obtained in Control mice to assess whether PRGF treatment could counteract the neurodegenerative action of NaIO_3_ on retinal tissue. The results observed in the present study showed that numerous deregulated proteins were related with two main processes, inflammation and fibrosis. The fibrosis of the retinal tissues, especially developed in the subretinal tissues, contributes to the progression of AMD, especially in advanced stages [43]. Myofibroblastic cells are mainly composed of scar tissue, which express different types of contractile filaments, such as alpha-smooth muscle actin (α-SMA) [44]. A recent study has shown that the systemic administration of NaIO_3_ induced an increase in SMA expression in retinal tissue, while intravitreal treatment of PRGF significantly reduced the expression of this protein in retinal tissue, but still at higher levels than in the Control group [22]. The results obtained in the present study show that several pathways related with the expression of SMA fibers, like Signaling by Rho Family GTPases; RHO GTPase cycle; Actin Cytoskeleton Signaling; and RHOGDI Signaling, among others, are upregulated after PRGF application in the mice systemically treated with NaIO_3_. These results suggest that a single application of PRGF may not be sufficient to reduce the fibrotic processes activated in the retina after the administration of NaIO_3_.

Although the pathogenic mechanism of AMD development is not fully understood, inflammation, together with oxidative stress, has been proposed as one of the most possible causes [45]. The retina is an immunoprivileged tissue, a status conferred by the blood–retinal barrier, local anti-inflammatory/anti-immune factors, and immune diversion associated with the anterior chamber. Loss of this privilege leads to abnormal immune and inflammatory activity, accelerating the pathogenesis of AMD [46]. The administration of NaIO_3_ also induces the retinal immune privilege loss, allowing the entry of immune cells from the subretinal tissues that accelerate the inflammatory processes related to AMD progression [47]. In the present study, the deregulated proteins of the mice retina treated with PRGF, compared to the Control group, are associated with pathways related to the inflammatory processes induced after systemic NaIO_3_ administration. This could imply that the inflammatory response triggered by NaIO_3_ may involve multiple activated pathways potentially reducing the PRGF’s effectiveness or that its efficacy is reduced in certain inflammatory pathways. However, the results demonstrated that the number of deregulated proteins and pathways associated with oxidative and cellular stress was significantly reduced after PRGF treatment when comparing the results obtained for PRGF vs. Control to the previous findings from Disease vs. Control. This suggests that PRGF has the capacity to mitigate the pathological effect of NaIO_3_ administration at this level. In addition, different pathways related to retinal homeostasis like Remodeling of Epithelial Adherens Junctions, Response to Elevated Platelet Cytosolic Ca^2+^, Selenoamino Acid Metabolism, and Caveolar-mediated Endocytosis Signaling were upregulated after PRGF treatment [48,49,50,51]. Although the application of PRGF did not lead to the complete homeostasis of retinal tissues after the administration of NaIO_3_, these results indicate that PRGF activates specific pathways to mitigate the degenerative effects induced by NaIO_3_ in the context of AMD.

The Ingenuity Pathway Analysis performed using the deregulated proteins obtained after comparing the PRGF and Disease groups showed that some relevant processes in AMD development, like angiogenesis and fibrosis, are in an equilibrium state after PRGF treatment. However, a previous study demonstrated that intravitreal administration of PRGF in a murine NaIO_3_ model reduced the expression of markers related to retinal fibrosis, like SMA and GFAP [22]. In addition, this study also showed that the PRGF injection did not induce retinal angiogenesis. Therefore, although several pathways related to angiogenesis induction are deregulated after PRGF administration, histological studies indicate that PRGF administration shifts the balance toward a non-angiogenic situation. In addition, it is important to highlight that two downregulated processes in the PRGF group are related not only to fibrosis but also to RPE degeneration, like Dopamine-DARPP32 Feedback in cAMP Signaling, and lipofuscin accumulation, like Calcium Signaling [52,53,54]. Both processes are closely associated with the development and progression of early-to-medium stages of AMD. These results strongly suggest that PRGF treatment mitigates the progression of AMD. Furthermore, diverse deregulated pathways state that PRGF treatment effectively mitigates the degenerative retinal effects induced by systemic NaIO_3_ administration. These hypotheses are also supported by the finding that several key processes in AMD development and progression, including inflammation, oxidative stress, and cellular stress, were reduced after PRGF administration. In addition, several canonical pathways related to the homeostasis overexpressed both in the PRGF group, such as LXR/RXR Activation and DHCR24 Signaling Pathway, and in the Disease group, such as Protein Kinase A Signaling and Opioid Signaling [55,56,57,58]. However, three of these pathways, including the DHCR24 Signaling Pathway, Protein Kinase A Signaling, and Opioid Signaling, are associated with a neuroprotective effect. This suggests that the retinal tissue is reacting to the stress induced by NaIO_3_ exposure and that the different retinal cells are activating diverse protective or compensatory signaling pathways in an effort to overcome severe oxidative and metabolic stress, reflecting a critical endogenous defense mechanism. Finally, it is important to highlight that PRGF application induced the activation of pathways like LXR/RXR Activation, which ameliorated lipid accumulation and oxidant-induced injury in RPE cells and decreased ocular inflammatory markers and lipid deposition [59]. These results suggest that PRGF treatment may reduce the accumulation of lipid deposits (drusen) in retinal tissues, a process essential for AMD development [7].

Although the findings were consistent, this study has some limitations that need to be considered. The AMD mouse model was obtained after a single NaIO_3_ administration, inducing an acute pathology that mimics the pathological mechanism of AMD [23]. The effect of this acute administration could be so high that a single administration of PRGF may not be sufficient to counterbalance the different deregulated processes following the acute administration of NaIO_3_. A recent study proposes different PRP application times to assess whether additional PRP applications induce a greater slowdown in the progression of AMD over time [19].

## 5. Conclusions

In summary, the results obtained in the present study demonstrated that systemic application of NaIO_3_-induced deregulation of several proteins and pathways in the retinal tissue that are related to AMD pathogenesis. In addition, although this is a preliminary study and new studies will be necessary, the findings obtained in the present study suggest that PRGF reduces the expression of different proteins related to AMD development and progression, indicating that PRGF could be a promising new therapeutic option to ameliorate AMD progression.

## Figures and Tables

**Table 1:** Canonical pathway analysis of the differentially expressed proteins in mice retina from Disease and Control groups. The 25 most significantly enriched canonical pathways (−log *p* values) are displayed. The z-score values obtained for each canonical pathway represent the magnitude to which a pathway is upregulated (positive value) or downregulated (negative value) in the Disease group relative to the Control group. The results are also clustered in functionally related groups of processes: inflammation; fibrosis; oxidative stress (Oxi. Stress); cellular stress (Cel. Stress); protein metabolism (Protein Metab.); energy metabolism (Energy Metab.); and angiogenesis.

Canonical Pathways	−log(*p*-Value)	z-Score	Related Processes
Neutrophil degranulation	33.20	5.67	Inflammation
Visual phototransduction	23.10	−5.21	-
Signaling by Rho Family GTPases	17.00	2.80	Fibrosis
EIF2 signaling	16.50	3.40	Oxi. Stress
Eukaryotic translation initiation	15.50	6.08	Cel. Stress
Selenoamino acid metabolism	15.10	5.33	Oxi. Stress
SRP-dependent cotranslational protein targeting to membrane	14.80	5.92	-
Response of EIF2AK4 (GCN2) to amino acid deficiency	14.20	5.39	Cel. Stress
Eukaryotic translation elongation	13.60	5.57	Protein Metab.
Nonsense-mediated decay (NMD)	13.40	5.48	Protein Metab.
Signaling by ROBO receptors	13.30	2.32	Angiogenesis
Mitochondrial protein degradation	13.00	−4.54	Energy Metab.
Sirtuin signaling pathway	11.90	2.12	Angiogenesis
Ribosomal quality control signaling pathway	11.50	5.34	Protein Metab.
Regulation of eIF4 and p70S6K signaling	11.50	2.65	Oxi. Stress
Class I MHC mediated antigen processing and presentation	11.40	5.08	Inflammation
Eukaryotic translation termination	11.30	5.29	Protein Metab.
RHO GTPase cycle	10.90	4.13	Fibrosis
IL-8 Signaling	10.80	3.05	Inflammation
Mitochondrial dysfunction	10.60	3.36	Energy Metab.
Major pathway of rRNA processing in the nucleolus and cytosol	10.30	5.15	Protein Metab.
CLEAR signaling pathway	9.29	2.59	Cel. Stress
Coronavirus pathogenesis pathway	8.78	−2.56	-
Protein ubiquitination pathway	8.54	2.14	Protein Metab.
Cristae formation	8.31	−3.61	Energy Metab.

**Table 2:** Canonical pathway analysis of the differentially expressed proteins in mice retina from PRGF and Control groups. The 25 most significantly enriched canonical pathways (−log *p* values) are displayed. The z-score values obtained for each canonical pathway represent the magnitude to which a pathway is upregulated (positive value) or downregulated (negative value) in the PRGF group relative to the Control group. The results are also clustered in functionally related groups of processes: inflammation; fibrosis; oxidative stress (Oxi. Stress); cellular stress (Cel. Stress); energy metabolism (Energy Metab.); angiogenesis; and homeostasis.

Canonical Pathways	−log(*p*-Value)	z-Score	Related Processes
Neutrophil Degranulation	38.60	5.44	Inflammation
Signaling by Rho Family GTPases	24.00	2.29	Fibrosis
RHO GTPase Cycle	23.40	4.08	Fibrosis
Actin Cytoskeleton Signaling	23.30	4.18	Fibrosis
Visual Phototransduction	23.10	−5.58	-
RHOGDI Signaling	21.60	−2.21	Fibrosis
Integrin Signaling	15.10	3.61	Inflammation
Class I MHC Mediated Antigen Processing and Presentation	13.30	3.16	Inflammation
Remodeling of Epithelial Adherens Junctions	13.20	2.11	Homeostasis
EIF2 Signaling	12.10	3.13	Oxi. stress
Regulation of Actin-based Motility by Rho	12.10	2.75	Fibrosis
ILK Signaling	11.40	2.97	Fibrosis
Striated Muscle Contraction	10.80	4.24	Fibrosis
RAC Signaling	10.60	3.02	Angiogenesis
Response to Elevated Platelet Cytosolic Ca^2+^	10.50	4.90	Homeostasis
Selenoamino Acid Metabolism	10.40	4.11	Homeostasis
fMLP Signaling in Neutrophils	9.72	2.12	Inflammation
CXCR4 Signaling	9.68	2.19	Inflammation
Caveolar-mediated Endocytosis Signaling	9.44	2.18	Homeostasis
Fcγ Receptor-mediated Phagocytosis in Macrophages and Monocytes	9.19	3.27	Inflammation
Paxillin Signaling	9.14	2.29	Fibrosis
Gluconeogenesis I	9.12	−2.31	Energy Metab.
Thrombin Signaling	8.93	2.41	Inflammation
Eukaryotic Translation Initiation	8.83	5.58	Cel. Stress
Leukocyte Extravasation Signaling	8.82	3.89	Inflammation

**Table 3:** Canonical pathway analysis of the differentially expressed proteins in mice retina from PRGF and Disease groups. The 25 most significantly enriched canonical pathways (−log *p* values) are displayed. The z-score values obtained for each canonical pathway represent the magnitude to which a pathway is upregulated (positive value) or downregulated (negative value) in the PRGF group relative to the Disease group. The results are mainly clustered in functionally related groups of processes: inflammation; fibrosis; oxidative stress (Oxi. Stress); cellular stress (Cel. Stress); energy metabolism (Energy Metab.); angiogenesis; and homeostasis.

Canonical Pathways	−log(*p*-Value)	z-Score	Related Processes
Striated Muscle Contraction	11.20	3.16	Cel migration
Calcium Signaling	11.10	−2.12	Inflammation, lipofucsin accumulation, and fibrosis
Protein Kinase A Signaling	10.50	−2.14	Homeostasis
Opioid Signaling	8.21	−2.71	Homeostasis
MHC class II Antigen Presentation	7.58	−3.46	Inflammation
LXR/RXR Activation	7.51	2.83	Homeostasis
DHCR24 Signaling Pathway	7.05	2.53	Homeostasis
Dilated Cardiomyopathy Signaling Pathway	6.79	−3.32	Fibrosis
Response to Elevated Platelet Cytosolic Ca^2+^	5.56	2.53	-
Hepatic Fibrosis Signaling Pathway	5.21	2.50	Fibrosis
Dopamine-DARPP32 Feedback in cAMP Signaling	5.20	−2.11	Fibrosis and RPE degeneration
Semaphorin Neuronal Repulsive Signaling Pathway	5.14	2.33	Angiogenesis
Smooth Muscle Contraction	5.07	2.45	Fibrosis
Nuclear Cytoskeleton Signaling Pathway	5.04	−2.31	Fibrosis
Corticotropin Releasing Hormone Signaling	4.94	−2.33	Inflammation and stress
Intra-Golgi and Retrograde Golgi-to-ER Traffic	4.64	−2.71	Protein Metab.
Eicosanoid Signaling	4.01	−2.11	Oxi. stress
Post-translational Protein Phosphorylation	3.66	2.65	-
Integration of Energy Metabolism	3.63	−2.65	Energy Metab.
HSP90 Chaperone Cycle for Steroid Hormone Receptors in the Presence of Ligand	3.33	−2.24	Cel. stress
Ephrin Receptor Signaling	3.29	−2.12	Angiogenesis
Regulation of Insulin-like Growth Factor (IGF) Transport and Uptake by IGFBPs	3.27	2.65	Angiogenesis
Synaptic Long-Term Potentiation	3.27	−2.65	-
Kinesins	3.16	−2.24	Protein Metab.
Formation of Fibrin Clot (Clotting Cascade)	3.01	2.00	Fibrosis

## Data Availability

The datasets used and/or analyzed during the current study are available from the corresponding author on reasonable request.

## Supplementary Materials

The following supporting information can be downloaded at: https://www.mdpi.com/article/10.3390/medicina61122235/s1, Supplementary File S1: Protein content data obtained from the different mouse belonging to the different groups (Control, Disease and PRGF); Supplementary File S2: Differential protein expression and Gene Ontology data from the comparison between the different groups; Supplementary File S3: Ingenuity Pathway Analysis (IPA) of deregulated proteins from the comparison between the different groups.

## Abbreviations

The following abbreviations are used in this manuscript:

AMD	Age-related macular degeneration
CB-PRP	Cord blood-derived platelet-rich plasma
DIA	Data-independent acquisition
FA	Formic acid
FASP	Filter-aided sample preparation
FDR	False discovery rate
GA	Geographic atrophy
GO	Gene ontology
IGF	Insulin-like growth factor
IPA	Ingenuity pathway analysis
NaIO_3_	Sodium iodate
nAMD	Neovascular AMD
PASEF	Parallel accumulation serial fragmentation
PBS	Phosphate buffered saline
PCA	Principal component analysis
PRGF	Plasma rich in growth factors
PRP	Platelet-rich plasma
ROS	Reactive oxygen species
RPE	Retinal pigment epithelium
α-SMA	Alpha-smooth muscle actin
